# The role of environmental impact in healthcare providers’ choices of inhalers for treatment of asthma and COPD: a discrete choice experiment

**DOI:** 10.1186/s12875-025-02941-8

**Published:** 2025-09-03

**Authors:** I. G. Arslan, M. Vervloet, E. W. de Bekker-Grob, K. Hek, B. J. Knottnerus, C. Wagner, L. van Eikenhorst

**Affiliations:** 1 Netherlands Institute for Health Services Research (Nivel), Utrecht, the Netherlands; 2 Erasmus School of Health Policy & Management, Erasmus University Rotterdam, Rotterdam, The Netherlands; 3 Erasmus Choice Modelling Centre, Erasmus University Rotterdam, Rotterdam, The Netherlands; 4 Erasmus Centre for Health Economics Rotterdam, Erasmus University Rotterdam, Rotterdam, The Netherlands; 5 Department of Public and Occupational Health, Amsterdam UMC, Vrije Universiteit Amsterdam, Amsterdam, the Netherlands

**Keywords:** Asthma, COPD, Climate change, Carbon footprint, Inhaler, General practice, Discrete choice experiment, Preferences

## Abstract

**Background:**

Pressurised metered-dose inhalers (pMDIs), often prescribed for the treatment of COPD and asthma have a high global warming potential (GWP) compared to dry powder inhalers (DPIs) and soft-mist inhalers. Despite calls to switch from high to DPIs or soft-mist inhalers, prescriptions of pMDIs have increased in recent years. Understanding healthcare providers' preferences can help develop strategies to promote prescribing low GWP inhalers. This study aimed to determine healthcare providers' preferences when prescribing inhalers for asthma and COPD, including its GWP (i.e. environmental impact).

**Methods:**

A survey containing a discrete choice experiment was conducted. Primary care providers were repetitively asked to choose between scenarios of inhalers that differed in five attributes: multidose or unidose system (i.e. ease of use), reduction in number of exacerbations, side effects, out of pocket costs and GWP. A multinomial logit model was used to determine the relative importance of the attributes.

**Results:**

A total of 76 healthcare providers (general practitioners (GPs), nurse specialists and nurse practitioners) completed the survey. On average, the attribute ‘reduction in number of exacerbations’ was most important in their choice for inhalers, followed by, ‘side effects’, ‘GWP’, ‘out of pocket costs’, and ‘multidose or unidose system’. Healthcare providers were willing to accept a high GWP inhaler instead of a low GWP inhaler when the inhaler reduced the number of exacerbations and were willing to opt for low GWP inhaler with higher out of pocket costs instead of a high GWP inhaler with lower out of pocket costs.

**Conclusions:**

Healthcare providers valued clinical factors (i.e. reduction in number of exacerbations and low side effects) as most important when choosing inhalers for their patients with COPD or asthma, however GWP was also an important driver of choice. Understanding these preferences can support strategies to support sustainable inhalers for COPD and asthma, contributing to climate change mitigation. For example by enhancing the provision of information regarding inhaler GWP, while ensuring that clinical effectiveness remains the primary focus.

**Supplementary Information:**

The online version contains supplementary material available at 10.1186/s12875-025-02941-8.

## Introduction

Climate change is an important threat to global health of our times, with the healthcare sector being a major contributor [1, 2]. Globally, the healthcare sector accounts for 4.4% of net CO₂ emissions, primarily driven by pharmaceuticals [3]. In the Netherlands, healthcare accounts for 8% of net CO₂ emissions [4]. Pressurised metered-dose inhalers (pMDIs), often prescribed for the treatment of Chronic Obstructive Pulmonary Disease (COPD) and asthma [5], contribute for 0.03% to the net global CO₂ emissions, mainly due to their propellants (HFA-134a/227) [6]. These inhalers have a higher global warming potential (GWP) compared to dry powder inhalers (DPIs) and soft-mist inhalers; the CO₂ equivalent footprint of a pMDI is up to 25 times greater [7, 8].

The British Thoracic Society recommends choosing a DPI when prescribing inhalers for newly diagnosed patients with COPD or asthma and when patients use multiple types of inhalers and can safely use a DPI, if the required medicine for the patient is available in a DPI [9]. Currently, 8.5% of the Dutch population uses inhalers of which more than half (55%) pMDIs [10]. Prescribing DPIs or soft-mist inhalers instead of pMDIs has the potential to reduce the CO₂ emissions, contributing to climate change mitigation [11–13]. The transition of 20 patients from pMDIs to DPIs or soft-mist inhalers can lead to emission reduction equivalent to replacing a conventional car with an electric vehicle [14]. Another benefit of prescribing DPIs or soft-mist inhalers instead of pMDIs is the indirect reduction of health risks for COPD and asthma patients as it helps mitigate the rise in summer smog and the increased exposure to pollen caused by climate change [14]. Important patient related conditions for switching to DPIs or soft-mist inhalers are sufficient inspiratory force generated by the patient and an adequate hand-lung coordination [15, 16]. Current user profiles reveal an opportunity for switching, given that approximately 180,000 patients in the Netherlands use both a DPIs or soft-mist inhalers and a pMDI [10]. In Sweden, only 13% of inhalers are pMDIs [17], compared to 55% in the Dutch population [10]. This demonstrates the possibility for safe and durable switching to DPIs or soft-mist inhalers, when patients receive adequate guidance from healthcare providers and the switch aligns with their needs and preferences.

In countries such as the Netherlands, the UK, and Scandinavian countries, prescribers in general practice play a crucial role in switching from pMDIs to DPIs or soft-mist inhalers, which primarily include general practitioners (GPs), but more recently also encompass nurse practitioners, physician assistants, nurse specialists and respiratory nurses [18, 19]. As they are often the first point of contact for patients, they typically make the initial decision regarding treatment and type of inhalers for COPD and asthma [20]. Recent guidelines from the Dutch College of General Practitioners recommend considering environmental impact when prescribing inhalers for asthma and COPD [14, 15]. Nevertheless, the number of pMDI prescriptions have increased in recent years [14].

Although various studies has been conducted internationally on the preferences and perspectives on inhalers [21–28], no study to date has examined the role of *environmental impact* of inhalers in the trade-offs that healthcare providers make when choosing a type of inhaler in COPD and asthma treatment. Understanding of this process can support strategies to promote environmentally friendly treatment for COPD and asthma. Therefore, our study aims to determine the preferences of healthcare providers for characteristics of inhalers for COPD and asthma, including environmental impact. This study focused on maintenance inhalers (taken on a regular daily basis) and not on short-acting inhalers (taken during acute symptoms) as this is an import part of long term treatment according to the guidelines of the Dutch College of General Practitioners for treatment of patients newly diagnosed with asthma or COPD [15, 16].

## Methods

### Discrete choice experiment

We used a discrete choice experiment (DCE) to determine the preferences of healthcare providers when prescribing maintenance inhalers for patients with asthma or COPD. A DCE is a technique used to quantify preferences for health goods, products and services [29]. In a DCE, it is assumed that preferences are based on the underlying characteristics of healthcare services, so-called attributes (e.g., out of pocket costs) [30]. Those attributes are specified by their attribute levels that refer to possible values (e.g., for out of pocket costs: €50,- or €100,-). We presented healthcare providers several alternatives of fictitious inhalers with different combinations of attribute levels, so-called choice tasks, in an online questionnaire. Healthcare providers were repeatedly asked to make a choice between two hypothetical alternatives. In this way, what matters to healthcare providers, how much it matters (i.e., relative importance) to healthcare providers, and how willing they are to give up on one attribute to gain something on another attribute (i.e. trade-offs) could be determined.

### Attributes and levels

We composed a list of potential attributes based on the results of our previous qualitative study on patients'and healthcare providers’ willingness to choose for low GWP inhalers [31] and from national guidelines for COPD and asthma [15, 16]. A literature review was conducted to complement this list. A search was conducted in PubMed database to identify qualitative studies, DCEs and literature reviews focusing on patients’ and healthcare providers’ experiences, preferences and perspectives on inhalers (Supplementary File 1 for the search strategy). The list covered the following domains: ease of use, hygiene, dose confirmation, reusable/disposable/recyclable, appearance and design, efficacy, side effects, costs, GWP, and patient satisfaction. In a DCE, the number of attributes to include is limited, as a higher number of attributes relates to a rising cognitive burden of the participant [32]. Therefore, we reduced the number of attributes by presenting the list of potential attributes to three GPs and asked them to rank the attributes from most to least important. We then selected the five attributes that were deemed most relevant by these GPs (Table 1), as attributes ranked sixth or higher were deemed substantially less important. Attribute levels were specified by publications of national sources [33], discussed within the research team and verified by the same GPs.

**Table 1:** Attributes and levels used in the discrete choice experiment

Attribute	Definition given to the participants in the survey	Levels
Multidose or unidose system (ease of use)	A'multidose'inhaler contains multiple doses within the inhaler. A'unidose'inhaler uses individual capsules and needs to be refilled before each inhalation. Therefore, a'unidose'inhaler always requires more steps before inhalation compared to a multidose system	• Unidose system • Multidose system
Reduction in number of exacerbations (flare-ups) per year (efficacy)	The use of inhaler can reduce the likelihood of exacerbations in the next year. This reduction varies depending on the type of inhaler and serves as an indicator of the effectiveness of the inhaler	• Likely to reduce one exacerbation in the upcoming year • Likely to reduce two exacerbations in the upcoming year • Likely to reduce three exacerbations in the upcoming year
Likelihood of side effects (safety)	The use of inhaler can cause side effects, such as dry mouth or palpitations. These side effects may lead to non-adherence to the agreed regimen	• Likely to experience no side effects • Likely to experience mild side effects, but they do not interfere with taking the medicine • Likely to experience moderate to severe side effects that may stop you from taking the medicine
Out of pocket costs	Annual costs the patient has to pay for using inhalers, not reimbursed by the health insurer	• €50,- per year • €150,- per year • €250,- per year
Global warming potential (GWP)	pMDIs contain greenhouse gases. The environmental impact of one inhalation from a pMDI, is 25 times higher than that of a low GWP inhaler (i.e. DPI or soft-mist inhaler). With average use of a pMDI (5.5 packages per year), the CO₂ equivalent emissions per year are equivalent to a round-trip flight from Amsterdam to Paris	• High GWP inhaler • Low GWP inhaler

### Experimental design

A D-efficient experimental design [34] with 18 choice tasks was designed using Ngene built within Survey Engine software [35] to maximize the statistical efficiency in measuring the main effects. In DCEs, the higher the number of choice tasks, the higher the cognitive burden for participants, which might impact the reliability of the results [32]. Therefore, we divided the design into two blocks of 9 choice tasks and randomly presented one of the two blocks to the participants [36]. One additional repeated choice task was included as a consistency check (i.e. internal validity) [37, 38]. We repeatedly asked the participants in each choice task what alternative they preferred for the hypothetical case presented in the DCE. Table 2 shows an example of a presented choice task included in the questionnaire.

**Table 2:** Example of a choice task

Imagine that you can choose between two different types of inhalers to prescribe to your patient, which of the following would you choose, Option A or Option B?		
	Option A	Option B
Multidose or unidose	Multidose	Unidose
Reduction in number of exacerbations (flare-ups) per year	Reduction of two exacerbations in the upcoming year	Reduction of two exacerbations in the upcoming year
Risk of side effects	Mild side effects but these do not affect the intake of medication	No side effects
Out of pocket costs per year	€150,-	€250,-
Global warming potential	High	Low
I would choose:		

To exclude contextual and patient-related factors that may influence the choice responses, such as insufficient lung capacity as a barrier for using any type of inhaler, participants were presented with a hypothetical patient case to base their choices on (Table 3).

**Table 3:** Hypothetical patient case presented in the questionnaire

“A patient with asthma or COPD for whom you are going to prescribe maintenance medication for the first time. This patient has sufficient lung capacity and coordination to use any type of inhaler no concomitant medications and an average socioeconomic status.”	

### Development of the questionnaire

The questionnaire also contained demographic questions, work-related questions and attitudinal questions about environmental impact after the choice tasks, based on the Theory of Planned Behaviour [39]. Participants were also asked if they were member of a professional organisation or network for environmentally friendly healthcare, such as ‘De Groene Huisarts’ (in English: ‘The Green GP’) [40]. The questionnaire was designed to take no longer than 10 min and included an explanation of the attributes and levels and a warm-up choice task before starting the choice tasks.

A draft version of the questionnaire was pre-tested with three GPs using cognitive interviews in which they were asked to read and think aloud while completing the questionnaire [41]. As a result, minor textual alterations to the questionnaire were made to improve the readability and face validity. Prior estimates of the attribute-levels were updated after a pilot run following good practices [34, 41]. The final version of the questionnaire translated from Dutch to English is presented in Supplementary File 2.

### Study population

Healthcare providers were approached through various channels: the individual network of the authors, general practices participating in the Nivel Primary Care Database [42] and general practices making use of the services of Calculus (a national company providing support to GPs with their declarations and administration). GPs, nurse practitioners, physician assistants, nurse specialists and respiratory nurses treating at least one COPD and/or asthma patient per week in general practice and gave informed consent were included. Ten gift vouchers worth €50,- each were raffled among participants who completed the survey.

### Statistical analyses

Descriptive characteristics were reported as means and standard deviations (SDs), medians and interquartile ranges (IQRs), and counts (n) and percentages (%), as appropriate.

Choice data were analysed using a multinomial logit model (Model A; multinomial logit model). The proportion of participants consistently answering the repeated choice task correctly was 89.5%, which suggest that respondents were largely providing stable answers, indicating high reliability. None of the participants always chose option A or B (i.e. flat-liners), which suggests a sufficient level of data quality. To avoid selection bias, no participants were a priori excluded from the analysis based on their answers to internal validity assessments [43]. We tested for linearity of the attributes. Out-of-pocket costs and number of exacerbations were coded as continuous variables, and multidose or unidose system, risk of side effects and GWP were treated as categorical variables, with the “worst” level (i.e. least desirable level) used as reference level.

In the second model, we included several interaction terms based on background characteristics with sufficient sample size within the subgroups to perform these analyses to assess whether the preferences for low GWP differed between: 1) GPs, and nurse practitioners and nurse specialists, 2) healthcare providers with over 10 years of experience in general practice and those with 10 or fewer years of experience, and 3) healthcare providers who were member of a professional organisation or network for environmentally friendly healthcare and those not (Model B; multinomial logit model plus systematic preference heterogeneity). The utility function used in the model is presented in Supplementary File 3.

Additional analyses were conducted to calculate the relative importance of each attribute in percentages by computing the difference between the minimum and maximum utility of each attribute divided by the sum of the differences between all utilities of all attributes (the higher the greater the importance relative to other attributes). We calculated the marginal rate of substitution (MRS), i.e. the willingness to pay for a low GWP inhaler and willingness to accept in number of reduction of exacerbation per year for a high GWP inhaler. Further information is provided in Supplementary File 4.

Statistical analyses were performed using R-Studio software version 4.3.1. For the analysis of choice data, Apollo package in R-Studio software was used [44].

## Results

### Characteristics of participants

A total of 76 participants completed the DCE questionnaire. Most of the participants were between 40–49 years old (28.9%) and female (75.0%) (Table 4). A total of 60.5% was working as a GP and the remaining 39.5% as a nurse practitioner/specialist in general practice. Most of the participants (68.4%) had over 10 years of working experience and almost half (48.7%) worked in a general practice located in an urban area. In addition, 10.5% was member of a professional organisation or network for environmentally friendly healthcare.

**Table 4:** Characteristics of participants

	***n* = 76**
	n (%)
Female	57 (75.0)
Age	
- 20–29 years	3 (3.9)
- 30–39 years	19 (25.0)
- 40–49 years	19 (25.0)
- 50–59 years	22 (28.9)
- ≥ 60 years	13 (17.1)
Profession	
- GP	46 (60.5)
- *Of which specialised in asthma/COPD*	9 (11.8)
- Nurse practitioner/nurse specialist	30 (39.5)
Working in general practice for	
- < 5 years	11 (14.5)
- 5–10 years	13 (17.1)
- > 10 years	52 (68.4)
Working in urban area	37 (48.7)
Member of a professional organisation or network for environmentally friendly healthcare	8 (10.5)

**Fig. 1:**
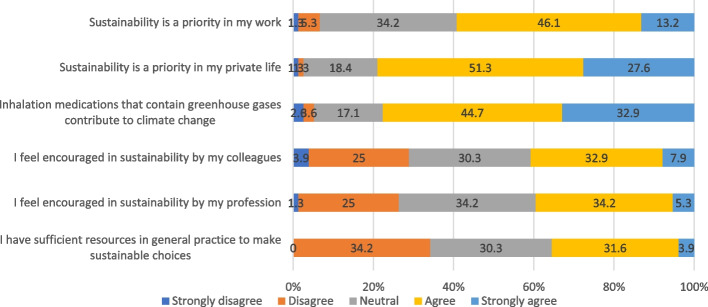
Attitudinal questions towards climate change’

A total of 59.3% of the participants (strongly) agreed that sustainability is a priority in their work, with an even higher percentage (78.9%) expressing this sentiment about their private lives (Fig. 1). The majority (77.6%) (strongly) agreed that inhalers containing greenhouse gases contribute to climate change. However, a smaller percentage (strongly) agreed in being encouraged by their profession colleagues (40.8%) or profession (39.5%) in sustainability and believed they had sufficient resources to make sustainable choices (35.5%) in general practice (i.e. external factors). There were differences in the attitude towards climate change between GPs and nurse practitioners/nurse specialists (see Supplementary File 5). In general, a higher percentage of GPs (strongly) agreed with all attitudinal questions (not statistically tested due to low sample size), especially in the sentiment that sustainability is a priority in their private lives (84.2% versus 73.7% respectively) and in being encouraged by their profession colleagues in sustainability (52.6% versus 29.0% respectively).

### Discrete choice experiment

Table 5 presents the preferences of healthcare providers (for utilities see Supplementary File 6). In general, all ORs were statistically significant, suggesting that all attributes played a role in their decision for inhalers, except for out of pocket costs. Healthcare providers preferred inhalers reducing the number of exacerbations with multidose system, no side effects and low GWP. GPs were more likely to choose low GWP inhalers compared to nurse practitioners/specialists (OR = 2.48; 95%CI = 1.50–4.09). Finally, we found no statistically significant interaction between preferences for a low GWP inhaler and years working in general practice or being member of a professional organisation or a network for environmentally friendly healthcare.

**Table 5:** Results of the multinomial logit model

**Attribute levels**	Model A**		Model B***	
	**OR**	**(95%CI)**	**OR**	**(95%CI)**
ASC*	**1.33**	**(1.10; 1.62)**	**1.32**	**(1.12; 1.55)**
Out of pocket costs per year (per €100,-)	**0.74**	**(0.62; 0.88)**	**0.72**	**(0.60; 0.87)**
Reduction in number of exacerbations per year (per one exacerbation)	**2.48**	**(2.08; 2.96)**	**2.51**	**(2.09; 3.02)**
*Multidose/unidose system*				
Unidose system *(reference level)*	1.00	-	1.00	
Multidose system	**1.34**	**(1.02; 1.74)**	**1.37**	**(1.02; 1.85)**
*Risk of side effects*				
No side effects *(reference level)*	1.00	-	1.00	
Mild side effects	**0.64**	**(0.43; 0.96)**	**0.66**	**(0.43; 0.99)**
Moderate to severe side effects	**0.10**	**(0.07; 0.14)**	**0.09**	**(0.06; 0.15)**
*Impact on CO₂ emissions*				
High GWP *(reference level)*	1.00	-	1.00	
Low GWP	**3.39**	**(2.56; 4.49)**	1.15	(0.96; 1.37)
*Systemic preference heterogeneity*				
> 10 years working in general practice x low GWP	-		1.58	(0.72; 3.45)
GP (excl. nurse practitioner/specialist) x low GWP	-		**2.48**	**(1.15; 5.36)**
Member of organisation or network for environmentally friendly healthcare x low GWP	-		0.87	(0.35; 2.18)
*AIC*	672.79		616.58	

Bold: Statistically significance at 5% level

*Abbreviations*: *OR* odds ratio, *CI* confidence interval, *ASC* alternative specific constant, *GP* general practitioner, *AIC* Akaike Information Criterion

^*^The alternative specific constant (ASC) took the left–right bias of the choice processes of the participants (i.e. participants always choosing option A or B) into account

^**^Model A includes the results of the multinomial logit model

^***^Model B includes the results of the multinomial logit model plus systematic preference heterogeneity with interaction terms

The coefficients from the models represent marginal utilities for different attribute levels and were converted from utilities to odds ratios (OR). A statistically significant OR (*p*-value < 0.05) indicates that the attribute level had an impact on the choice process of the participants. If the OR for an attribute level is higher than one, this indicates that this level is preferred over the reference level of the same attribute. The higher the OR, the greater the preference. For a correct interpretation of the results, it is important to notice the different units of measurement, e.g. out of pocket costs is a continuous variable that is measured per euro, whereas multidose system is a categorical variable that are compared to their reference level

**Fig. 2:**
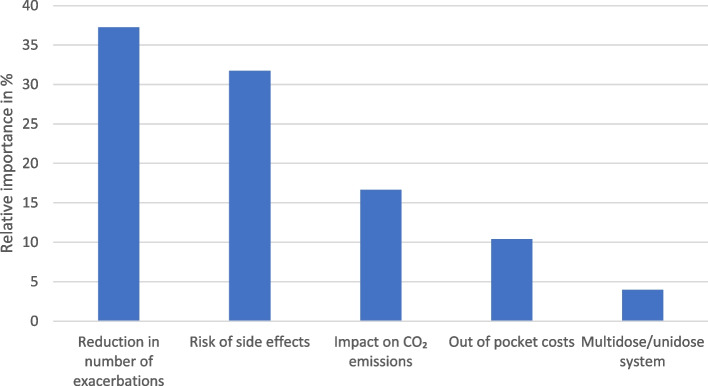
Relative importance of the attributes’

Figure 2 shows the relative importance of the attributes. The reduction in number of exacerbations and the risk of side effects were most important relative to all other attributes. This was followed by impact on CO₂ emissions, and out of pocket costs and multidose/unidose system were least important.

### Marginal rates of substitution

Healthcare providers were willing to accept a high GWP inhaler instead of a low GWP inhaler when the inhaler reduced the number of exacerbations. In addition, they were willing to opt for an inhaler with higher out of pocket costs in exchange for a low GWP instead of a high GWP inhaler.

## Discussion

### Main findings

This study demonstrates that while healthcare providers valued clinical factors (i.e. reduction in number of exacerbations and low side effects) as most important when choosing inhalers for their patients with COPD or asthma, low global warming potential (GWP) was also an important driver of choice. Out of pocket costs for the patient and multidose/unidose system of the inhaler were also found to be drivers of choice, but they were deemed less important. Our findings further indicate that healthcare providers are willing to choose for high GWP inhalers over low GWP alternatives if the inhaler reduced the number of exacerbations even with higher out of pocket costs.

### Comparison with previous research

Findings from previous DCEs on preferences for inhalers for COPD and asthma, which included both clinical and system characteristics of inhalers, correspond to our result that healthcare providers attach more importance to clinical characteristics (e.g. effectiveness) than system characteristics (e.g. cost attributes) [24]. However, no DCE to date has investigated the role of *environmental impact* of inhalers relative to these clinical and system characteristics in healthcare providers’ choices. A recent qualitative study reported that healthcare providers prioritize clinical efficacy over the environmental impact of inhalers, which is supported by the findings of the current study [45]. Furthermore, this study showed that while most healthcare providers value environmentally sustainable practices both in their work and private lives, only a minority feels encouraged or adequately resourced to make sustainable choices in general practice. Resource limitations are frequently reported as barriers to implementing sustainable practices in healthcare [46] and should be addressed in future initiatives for facilitating sustainable practices (e.g. stock and accessibility of low GWP inhalers).

### Implications for practice and policy

The introduction of novel pMDIs with greener propellants expected in 2025 may reduce the environmental impact of inhalers, but continued efforts to promote prescribing low GWP inhalers will remain essential as high GWP inhalers continue to be available on the market. This study shows that healthcare providers are willing to consider the GWP of inhalers when prescribing inhalers. Prescribing low GWP inhalers to newly diagnosed patients and switching current high GWP inhaler users to low GWP alternatives in patients with sufficient inspiratory force and an adequate hand-lung coordination could contribute to meet this target. Previous research shows that healthcare providers with adequate knowledge of sustainable prescribing are more likely to prescribe sustainable medications [47]. Displaying the GWP or integrating low-GWP options as the default choice in electronic prescribing systems used in general practice may encourage the adoption of low GWP inhalers. Recent research indicated that patients are willing to choose more sustainable healthcare options, even when these may be less effective [48]. However, they often feel inadequately informed by their healthcare providers. Educating and informing healthcare providers and patients about medication switching, communication to patients throughout this process and shared-decision making between the healthcare provider and patient can support the choice for low GWP inhalers. Also, addressing patient knowledge on sustainability is essential, as patient awareness of sustainable medication appears to be low [46, 49]. Another strategy to promote low GWP inhalers can be providing feedback to healthcare providers on their prescribing behaviour based on electronic health record data, as this approach has been proven to be effective for appropriate prescribing behaviour [50]. Regardless of the treatment decision, the selected treatment should adequately meet patients’ needs and preferences in terms of effectiveness and usability, while also balancing environmental considerations [51].

### Strengths and limitations of this study

The use of a DCE design is a strength of this study, as it is a robust and well-established method for capturing and quantifying preferences in decision-making [32, 34] Furthermore, our sample is generally representative of the age and sex distribution of GPs in the Netherlands [52]. Another strength of this study is included a diverse range of primary care inhaler prescribers, which enhances the representativeness of the findings. However, due to the low sample size, we were not able to perform more complex analyses that could reveal additional information on preference heterogeneity. Also, for attribute selection, we used findings from our previous qualitative study on preferences for low-GWP inhalers, national COPD and asthma guidelines, and conducted a literature review of qualitative studies and DCEs. We then asked three GPs to rank the attributes by importance and selected the five most relevant for inclusion in the DCE. A limitation of this process is that nurses—who made up 39.5% of the respondents—were not involved in the ranking process. Including their input might have improved the representativeness and practical relevance of the attribute selection. Lastly, findings of this study represent choices in hypothetical settings that may not fully correspond to choices in real life, although recent research has shown that DCEs are able to predict choices [53]. Preferences may vary depending on context, such as country-specific differences in healthcare systems. Preferences may differ for more complex patients than presented in the current hypothetical scenario, where for example the attribute multidose versus unidose systems (i.e. ease of use) could be more important.

### Further research

This study showed that GPs were more likely to choose low GWP inhalers compared to nurse practitioners and specialists. Future research exploring differences in preferences among healthcare provider groups is needed to help tailor strategies to promote low GWP-inhalers. Additionally, this study demonstrated that out-of-pocket costs are a relatively less important drivers of choice for healthcare providers compared to other attributes, which may differ for patients. Investigating patient preferences for inhalers including its GWP would provide valuable insights into potential differences between patient and provider priorities, supporting shared decision-making in prescribing low GWP inhalers.

## Conclusions

In conclusion, healthcare providers valued clinical factors (i.e. reduction in number of exacerbations and low side effects) as most important when choosing inhalers for their patients with COPD or asthma, however GWP was also an important driver of choice. They appear willing to make more sustainable choices, despite feeling constrained by external factors such as limited resources. These insights can support strategies to promote the use of low GWP inhalers for the treatment of COPD and asthma, contributing to climate change mitigation. For example by enhancing the provision of information regarding inhaler GWP, while ensuring that clinical effectiveness remains the primary focus.

## Supplementary Information

Supplementary Material 1.

Supplementary Material 2.

Supplementary Material 3.

Supplementary Material 4.

Supplementary Material 5.

Supplementary Material 6.

## Data Availability

The aggregated data are available on request from the corresponding author.
