# Supplementary material for: The role of environmental impact in healthcare providers’ choices of inhalers for treatment of asthma and COPD: a discrete choice experiment
Source: BMC Prim Care. 2025 Sep 3;26:278. doi: 10.1186/s12875-025-02941-8 (PMC12406421; doi:10.1186/s12875-025-02941-8)
Supplement: Supplementary file 1 — Supplementary Material 1. [file 12875_2025_2941_MOESM1_ESM.docx]

**SUPPLEMENTARY FILE 1**

**Search strategy in Pubmed for identifying potential attributes**

(Asthma [Mesh] OR COPD [tiab] OR chronic obstructive airways disease [tiab] OR respiratory disease [tiab] OR ((pulmonary disease, chronic obstructive) [Mesh]) AND (inhaler* [tiab] OR (metered dose inhaler [Mesh]) OR (dry powder inhalers [Mesh])) AND (patient perspective* [tiab] OR healthcare provider perspective* [tiab] OR user perspective* [tiab] OR patient experience* [tiab] OR healthcare provider experience* [tiab] OR user experience* [tiab] OR healthcare provider preference* [tiab] OR (patient preference [Mesh]) OR user preference* [tiab] OR attribute* [tiab] OR utilit* [tiab] OR (patient satisfaction [Mesh])) AND (interview [tiab] OR qualitative [tiab] OR focus group* [tiab] discrete choice experiment [tiab] OR DCE [tiab] OR survey [tiab] OR questionnaire [tiab] OR conjoint [tiab])

The search strategy was restricted to papers published from 1 September 2010 to 2023 and published in English or Dutch, including human population (excluding animal studies).
