# Supplementary material for: The role of environmental impact in healthcare providers’ choices of inhalers for treatment of asthma and COPD: a discrete choice experiment
Source: BMC Prim Care. 2025 Sep 3;26:278. doi: 10.1186/s12875-025-02941-8 (PMC12406421; doi:10.1186/s12875-025-02941-8)
Supplement: Supplementary file 2 — Supplementary Material 2. [file 12875_2025_2941_MOESM2_ESM.docx]

**SUPPLEMENTARY FILE 2**

**Explanation of the questionnaire**

Thank you for participating in this study! We start with some general questions followed by questions about your preferences regarding inhalation medication for asthma and COPD. Please complete the questionnaire as thoroughly as possible.

**General questions**

1. What is your gender?

- Male
- Female
- Prefer not to answer
- Other, namely: ….

1. What is your age?

- 20-29 years
- 30-39 years
- 40-49 years
- 50-59 years
- ≥60 years

1. What is your profession?

- General practitioner
- Dispensing general practitioner
- Nurse practitioners
- Nurse specialist
- Physician assistant
- Respiratory nurse
- Other [exclusion; not eligible, proceed to ‘Closure after screening question (1)’]

1. How many patients do you typically see per week in your practice for asthma and/or COPD?

- 0
- 1-5
- 6-10
- 11-15
- >15

[If 0: exclusion, not eligible, proceed to ‘Closure after screening question (2)’]

**Closure after Screening Question (1)**

Thank you for your participation. You have indicated that you have another profession. Therefore, this questionnaire is not relevant to you. The questionnaire will be terminated. If you have any questions, please contact XXX [contact details of researchers].

[End of questionnaire]

**Closure after Screening Question (2)**

Thank you for your participation. You have indicated that you see (almost) no patients with asthma and/or COPD in your practice. Therefore, this questionnaire is not relevant to you. The questionnaire will be terminated. If you have any questions, please contact XXX [contact details of researchers].

[End of questionnaire]

In the next section, we will ask you to choose between different characteristics of inhalation medication for asthma and COPD. Below, we first provide an explanation of the five characteristics covered in this study. It is important to read this carefully before proceeding to the example.

**Multidose system:** A 'multidose' inhaler contains multiple doses within the device. In contrast, a 'unidose' inhaler uses separate capsules containing medication the patient must insert before each use. Therefore, a 'unidose' inhaler always requires more handling than a multidose system.

**Reduction in the number of exacerbations:** The use of inhalation medication can reduce the risk of exacerbations in the upcoming year. This reduction may vary among different inhalation medications and serves as an indicator of its effectiveness.

**Risk of side effects:** Inhalation medication can cause side effects such as a dry mouth or heart palpitations. These side effects may lead to non-adherence of medication use.

**Out-of-pocket costs:** Costs that the patient must pay themselves for healthcare (not covered by the health insurer), regardless of whether they have reached their deductible.

**Impact on CO₂ emissions:** Pressurised metered-dose inhalers (pMDIs) contain greenhouse gases. The environmental impact of a single pMDI is 25 times greater compared to a dry powder inhaler due to the propellant gas. With an average use of a pMDI (5.5 packages per year), the CO₂-equivalent emissions per year are equal to a round-trip flight from Amsterdam to Paris.

**Choice sets**

**Below, we present several options that look similar but differ slightly each time. We ask you to choose between these options, as this is necessary for us to understand your preferences. For each choice, indicate your preference for a patient with asthma or COPD. There are no right or wrong answers.**

**We understand that some situations may differ from those in your current work environment. Please try to imagine the proposed scenario as accurately as possible. You do not need to consider your own logistical or financial factors.**

**We also recognize that your choice of inhalation medication may vary depending on the patient. Therefore, we ask you to base your responses on the following case:**

A patient with asthma or COPD for whom you are going to prescribe maintenance medication for the first time. This patient has sufficient lung capacity and coordination to use any type of inhaler, no concomitant medications, and an average socioeconomic status.

Below is an example choice set (you do not need to answer this question):

Imagine that you can choose between two different types of inhalers to prescribe to your patient, which of the following would you choose, Option A or Option B?

|  | Option A | Option B |
| --- | --- | --- |
| Multidose or unidose | Multidose | Unidose |
| Reduction in number of exacerbations (flare-ups) per year | Likely to reduce **two** exacerbations in the upcoming year | Likely to reduce **two** exacerbations in the upcoming year |
| Risk of side effects | Likely to experience **mild** side effects, but they do **not** interfere with taking the medicine | Likely to experience **no** side effects |
| Out of pocket costs per year | €150,- | €250,- |
| Global warming potential | High | Low |
| I would choose: | X |  |

In this case, Option A has been chosen. This option is a multidose inhaler. Use of this inhaler reduces the risk of **two** exacerbations in the upcoming year and causes mild side effects but these do **not** affect the intake of medication. The annual out-of-pocket cost for the inhaler is €**150**. The inhaler contains has a **high** global warming potential.

**Next, you will complete 5 choice sets, followed by a few background questions. After that, you will complete another 5 choice sets.**

[To limit the number of choice tasks for participants, two blocks of choice tasks were presented. Participant were randomly allocated to either block A or block B of the choice tasks. Each participant had to complete a total of 10 choice tasks.]

1a. Imagine that you can choose between two different types of inhalers to prescribe to your patient, which of the following would you choose, Option A or Option B?

|  | Option A | Option B |
| --- | --- | --- |
| Multidose or unidose | Unidose | Multidose |
| Reduction in number of exacerbations (flare-ups) per year | Likely to reduce **three** exacerbations in the upcoming year | Likely to reduce **one** exacerbation in the upcoming year |
| Risk of side effects | Likely to experience **moderate to severe** side effects that may stop you from taking the medicine | Likely to experience **no** side effects |
| Out of pocket costs per year | €50,- | €250,- |
| Global warming potential | High | Low |
| I would choose: |  |  |

1b. Imagine that you can choose between two different types of inhalers to prescribe to your patient, which of the following would you choose, Option A or Option B?

|  | Option A | Option B |
| --- | --- | --- |
| Multidose or unidose | Multidose | Unidose |
| Reduction in number of exacerbations (flare-ups) per year | Likely to reduce **one** exacerbations in the upcoming year | Likely to reduce **one** exacerbation in the upcoming year |
| Risk of side effects | Likely to experience **no** side effects | Likely to experience **moderate to severe** side effects that may stop you from taking the medicine |
| Out of pocket costs per year | €150,- | €250,- |
| Global warming potential | High | Low |
| I would choose: |  |  |

2a. Imagine that you can choose between two different types of inhalers to prescribe to your patient, which of the following would you choose, Option A or Option B?

|  | Option A | Option B |
| --- | --- | --- |
| Multidose or unidose | Unidose | Multidose |
| Reduction in number of exacerbations (flare-ups) per year | Likely to reduce **two** exacerbations in the upcoming year | Likely to reduce **one** exacerbation in the upcoming year |
| Risk of side effects | Likely to experience **no** side effects | Likely to experience moderate to severe side effects that **may stop** you from taking the medicine |
| Out of pocket costs per year | €150,- | €250,- |
| Global warming potential | High | Low |
| I would choose: |  |  |

2b. Imagine that you can choose between two different types of inhalers to prescribe to your patient, which of the following would you choose, Option A or Option B?

|  | Option A | Option B |
| --- | --- | --- |
| Multidose or unidose | Unidose | Multidose |
| Reduction in number of exacerbations (flare-ups) per year | Likely to reduce **two** exacerbations in the upcoming year | Likely to reduce **three** exacerbation in the upcoming year |
| Risk of side effects | Likely to experience mild side effects, but they **do not interfere** with taking the medicine | Likely to experience moderate to severe side effects that **may stop** you from taking the medicine |
| Out of pocket costs per year | €250,- | €150,- |
| Global warming potential | Low | High |
| I would choose: |  |  |

3a. Imagine that you can choose between two different types of inhalers to prescribe to your patient, which of the following would you choose, Option A or Option B?

|  | Option A | Option B |
| --- | --- | --- |
| Multidose or unidose | Unidose | Multidose |
| Reduction in number of exacerbations (flare-ups) per year | Likely to reduce **one** exacerbation in the upcoming year | Likely to reduce **three** exacerbations in the upcoming year |
| Risk of side effects | Likely to experience mild side effects, but they **do not interfere** with taking the medicine | Likely to experience mild side effects, but they **do not interfere** with taking the medicine |
| Out of pocket costs per year | €50,- | €250,- |
| Global warming potential | Low | High |
| I would choose: |  |  |

3b. Imagine that you can choose between two different types of inhalers to prescribe to your patient, which of the following would you choose, Option A or Option B?

|  | Option A | Option B |
| --- | --- | --- |
| Multidose or unidose | Multidose | Unidose |
| Reduction in number of exacerbations (flare-ups) per year | Likely to reduce **two** exacerbation in the upcoming year | Likely to reduce **one** exacerbations in the upcoming year |
| Risk of side effects | Likely to experience moderate to severe side effects that **may stop** you from taking the medicine | Likely to experience **no** side effects |
| Out of pocket costs per year | €50,- | €150,- |
| Global warming potential | Low | High |
| I would choose: |  |  |

4a. Imagine that you can choose between two different types of inhalers to prescribe to your patient, which of the following would you choose, Option A or Option B?

|  | Option A | Option B |
| --- | --- | --- |
| Multidose or unidose | Multidose | Multidose |
| Reduction in number of exacerbations (flare-ups) per year | Likely to reduce **two** exacerbations in the upcoming year | Likely to reduce **one** exacerbation in the upcoming year |
| Risk of side effects | Likely to experience mild side effects, but they **do not interfere** with taking the medicine | Likely to experience **no** side effects |
| Out of pocket costs per year | €150,- | €50,- |
| Global warming potential | High | Low |
| I would choose: |  |  |

4b. Imagine that you can choose between two different types of inhalers to prescribe to your patient, which of the following would you choose, Option A or Option B?

|  | Option A | Option B |
| --- | --- | --- |
| Multidose or unidose | Multidose | Unidose |
| Reduction in number of exacerbations (flare-ups) per year | Likely to reduce **one** exacerbations in the upcoming year | Likely to reduce **two** exacerbation in the upcoming year |
| Risk of side effects | Likely to experience **no** side effects | Likely to experience moderate to severe side effects that **may stop** you from taking the medicine |
| Out of pocket costs per year | €50,- | €150,- |
| Global warming potential | High | Low |
| I would choose: |  |  |

5a. Imagine that you can choose between two different types of inhalers to prescribe to your patient, which of the following would you choose, Option A or Option B?

|  | Option A | Option B |
| --- | --- | --- |
| Multidose or unidose | Multidose | Unidose |
| Reduction in number of exacerbations (flare-ups) per year | Likely to reduce **two** exacerbations in the upcoming year | Likely to reduce **three** exacerbations in the upcoming year |
| Risk of side effects | Likely to experience mild side effects, but they **do not interfere** with taking the medicine | Likely to experience mild side effects, but they **do not interfere** with taking the medicine |
| Out of pocket costs per year | €50,- | €250,- |
| Global warming potential | Low | High |
| I would choose: |  |  |

5b. Imagine that you can choose between two different types of inhalers to prescribe to your patient, which of the following would you choose, Option A or Option B?

|  | Option A | Option B |
| --- | --- | --- |
| Multidose or unidose | Multidose | Unidose |
| Reduction in number of exacerbations (flare-ups) per year | Likely to reduce **one** exacerbations in the upcoming year | Likely to reduce **two** exacerbations in the upcoming year |
| Risk of side effects | Likely to experience moderate to severe side effects that **may stop** you from taking the medicine | Likely to experience **no** side effects |
| Out of pocket costs per year | €250,- | €150,- |
| Global warming potential | High | Low |
| I would choose: |  |  |

**Now, a few background questions will follow.**

1. Are you a specialized general practitioner in asthma/COPD?

- Yes
- No

1. How long have you been practicing in a general practice?

- <5 years
- 5-10 years
- >10 years

1. What type of organization do you work?

- Duo-practice
- Group practice
- Solo-practice
- HOED (independent general practitioners in a single practice)
- AHOED (pharmacy and general practitioner(s) within the same building)
- Other, namely; …………………………………

1. Do you practice in an urban or rural area?

- Urban area
- Rural area

**Now, a few background questions will follow. Now, the last 5 choice sets will follow. You can base your responses on the same case as before:**

**Now, the last 5 choice sets will follow. You can base your responses on the same case as before:**

A patient with asthma or COPD for whom you are going to prescribe maintenance medication for the first time. This patient has sufficient lung capacity and coordination to use any type of inhaler, no concomitant medications, and an average socioeconomic status.

6a. Imagine that you can choose between two different types of inhalers to prescribe to your patient, which of the following would you choose, Option A or Option B?

|  | Option A | Option B |
| --- | --- | --- |
| Multidose or unidose | Multidose | Unidose |
| Reduction in number of exacerbations (flare-ups) per year | Likely to reduce **three** exacerbations in the upcoming year | Likely to reduce **three** exacerbations in the upcoming year |
| Risk of side effects | Likely to experience **no** side effects | Likely to experience mild side effects, but they **do not interfere** with taking the medicine |
| Out of pocket costs per year | €250,- | €50,- |
| Global warming potential | Low | High |
| I would choose: |  |  |

6b. Imagine that you can choose between two different types of inhalers to prescribe to your patient, which of the following would you choose, Option A or Option B?

|  | Option A | Option B |
| --- | --- | --- |
| Multidose or unidose | Unidose | Multidose |
| Reduction in number of exacerbations (flare-ups) per year | Likely to reduce **three** exacerbations in the upcoming year | Likely to reduce **two** exacerbations in the upcoming year |
| Risk of side effects | Likely to experience **no** side effects | Likely to experience mild side effects, but they **do not interfere** with taking the medicine |
| Out of pocket costs per year | €250,- | €150,- |
| Global warming potential | Low | High |
| I would choose: |  |  |

7a. Imagine that you can choose between two different types of inhalers to prescribe to your patient, which of the following would you choose, Option A or Option B?

|  | Option A | Option B |
| --- | --- | --- |
| Multidose or unidose | Unidose | Unidose |
| Reduction in number of exacerbations (flare-ups) per year | Likely to reduce **three** exacerbations in the upcoming year | Likely to reduce **one** exacerbation in the upcoming year |
| Risk of side effects | Likely to experience moderate to severe side effects **that may stop** you from taking the medicine | Likely to experience **no** side effects |
| Out of pocket costs per year | €250,- | €50,- |
| Global warming potential | Low | Low |
| I would choose: |  |  |

7b. Imagine that you can choose between two different types of inhalers to prescribe to your patient, which of the following would you choose, Option A or Option B?

|  | Option A | Option B |
| --- | --- | --- |
| Multidose or unidose | Unidose | Multidose |
| Reduction in number of exacerbations (flare-ups) per year | Likely to reduce **three** exacerbations in the upcoming year | Likely to reduce **two** exacerbation in the upcoming year |
| Risk of side effects | Likely to experience **no** side effects | Likely to experience mild side effects, but they **do not interfere** with taking the medicine |
| Out of pocket costs per year | €150,- | €50,- |
| Global warming potential | Low | High |
| I would choose: |  |  |

8a. Imagine that you can choose between two different types of inhalers to prescribe to your patient, which of the following would you choose, Option A or Option B?

|  | Option A | Option B |
| --- | --- | --- |
| Multidose or unidose | Unidose | Multidose |
| Reduction in number of exacerbations (flare-ups) per year | Likely to reduce **one** exacerbation in the upcoming year | Likely to reduce **two** exacerbations in the upcoming year |
| Risk of side effects | Likely to experience mild side effects, but they **do not interfere** with taking the medicine | Likely to experience moderate to severe side effects **that may stop** you from taking the medicine |
| Out of pocket costs per year | €250,- | €150,- |
| Global warming potential | High | Low |
| I would choose: |  |  |

8b. Imagine that you can choose between two different types of inhalers to prescribe to your patient, which of the following would you choose, Option A or Option B?

|  | Option A | Option B |
| --- | --- | --- |
| Multidose or unidose | Multidose | Unidose |
| Reduction in number of exacerbations (flare-ups) per year | Likely to reduce **three** exacerbation in the upcoming year | Likely to reduce **two** exacerbations in the upcoming year |
| Risk of side effects | Likely to experience mild side effects, but they **do not interfere** with taking the medicine | Likely to experience mild side effects, but they **do not interfere** with taking the medicine |
| Out of pocket costs per year | €50,- | €250,- |
| Global warming potential | High | Low |
| I would choose: |  |  |

9a. Imagine that you can choose between two different types of inhalers to prescribe to your patient, which of the following would you choose, Option A or Option B?

|  | Option A | Option B |
| --- | --- | --- |
| Multidose or unidose | Multidose | Unidose |
| Reduction in number of exacerbations (flare-ups) per year | Likely to reduce **one** exacerbation in the upcoming year | Likely to reduce **three** exacerbations in the upcoming year |
| Risk of side effects | Likely to experience moderate to severe side effects **that may stop** you from taking the medicine | Likely to experience moderate to severe side effects **that may stop** you from taking the medicine |
| Out of pocket costs per year | €150,- | €50,- |
| Global warming potential | Low | High |
| I would choose: |  |  |

9b. Imagine that you can choose between two different types of inhalers to prescribe to your patient, which of the following would you choose, Option A or Option B?

|  | Option A | Option B |
| --- | --- | --- |
| Multidose or unidose | Unidose | Multidose |
| Reduction in number of exacerbations (flare-ups) per year | Likely to reduce **one** exacerbation in the upcoming year | Likely to reduce **three** exacerbations in the upcoming year |
| Risk of side effects | Likely to experience moderate to severe side effects **that may stop** you from taking the medicine | Likely to experience **no** side effects |
| Out of pocket costs per year | €150,- | €50,- |
| Global warming potential | High | High |
| I would choose: |  |  |

10a. Imagine that you can choose between two different types of inhalers to prescribe to your patient, which of the following would you choose, Option A or Option B?

|  | Option A | Option B |
| --- | --- | --- |
| Multidose or unidose | Unidose | Multidose |
| Reduction in number of exacerbations (flare-ups) per year | Likely to reduce **three** exacerbations in the upcoming year | Likely to reduce **one** exacerbation in the upcoming year |
| Risk of side effects | Likely to experience moderate to severe side effects **that may stop** you from taking the medicine | Likely to experience **no** side effects |
| Out of pocket costs per year | €50,- | €250,- |
| Global warming potential | High | Low |
| I would choose: |  |  |

10b. Imagine that you can choose between two different types of inhalers to prescribe to your patient, which of the following would you choose, Option A or Option B?

|  | Option A | Option B |
| --- | --- | --- |
| Multidose or unidose | Multidose | Unidose |
| Reduction in number of exacerbations (flare-ups) per year | Likely to reduce **one** exacerbations in the upcoming year | Likely to reduce **one** exacerbation in the upcoming year |
| Risk of side effects | Likely to experience **no** side effects | Likely to experience **moderate to severe** side effects that may stop you from taking the medicine |
| Out of pocket costs per year | €150,- | €250,- |
| Global warming potential | High | Low |
| I would choose: |  |  |

**Now a few questions will follow about your attitude towards climate topics.**

1. Indicate to what extent you agree with the following statements:

|  | Strongly disagree | Disagree | Neutral | Agree | Strongly agree |
| --- | --- | --- | --- | --- | --- |
| Sustainability is a priority in my work |  |  |  |  |  |
| Sustainability is a priority in my private life |  |  |  |  |  |
| Inhalation medication containing greenhouse gases contributes to climate change |  |  |  |  |  |
| I feel encouraged in sustainability by my colleagues |  |  |  |  |  |
| I feel encouraged in sustainability by my profession |  |  |  |  |  |
| I have sufficient resources (such as financial recourses, time and personel capacity) in my general practice to make sustainable choices |  |  |  |  |  |

11. Have you joined a movement for sustainable general practitioners (such as ”De Groene Huisarts”)?

- Yes
- No
- I don’t know

**Below are a few final questions.**

1. How difficult did you find it to complete this questionnaire?

One (1) means 'very easy' and five (5) means 'very difficult'

1 2 3 4 5

2. How long did you find this questionnaire?

One (1) means 'short' and three (3) means 'long'

1 2 3

3. Do you have any comments?

....................................................................................................................................................................................................................................................................................................................................................................................................................................................................................................................................................................................................................................................................................................

4. Would you like to receive the conclusion of this study by email after completion?

- Yes, please send it to the following email address:

…………………………………………………………………………………………………...

- No

5. Would you like a chance to win a €50 gift card?

- Yes, I would like to be notified via the following email address: ………………………….
- No

**This is the end of the questionnaire. Thank you very much for completing it! If you have any questions about the study, please contact XXX [contact details of researchers].**

Click **'Next'** to complete the questionnaire.
