# Supplementary material for: The role of environmental impact in healthcare providers’ choices of inhalers for treatment of asthma and COPD: a discrete choice experiment
Source: BMC Prim Care. 2025 Sep 3;26:278. doi: 10.1186/s12875-025-02941-8 (PMC12406421; doi:10.1186/s12875-025-02941-8)
Supplement: Supplementary file 3 — Supplementary Material 3. [file 12875_2025_2941_MOESM3_ESM.docx]

**SUPPLEMENTARY FILE 3**

**Utility functions**

$${V\left( alt1 \right)}_{nsj}= \beta_{0} + \beta_{1} {out of pocket costs}_{\mathrm{nsj}}+\beta_{2} {reduction of two of exacerbations}_{\mathrm{nsj}}+\beta_{3} {reduction of three exacerbations}_{\mathrm{nsj}} + \beta_{4}{{mild side effects}_{\mathrm{nsj}} + \beta_{5} moderate to severe side effects}_{\mathrm{nsj}}+ \beta_{6}\mathrm{multidose}{}_{\mathrm{nsj}}+ \beta_{7} {low GWP}_{\mathrm{nsj}}+\beta_{8} {low GWP}_{\mathrm{nsj}} high\_work\_experience+ \beta_{9} {low GWP}_{\mathrm{nsj}} \mathrm{GP} + \beta_{10} {low GWP}_{\mathrm{nsj}} environment\_member$$

$${V\left( alt2 \right)}_{nsj}= \beta_{1} {out of pocket costs}_{\mathrm{nsj}}+\beta_{2} {reduction of two of exacerbations}_{\mathrm{nsj}}+\beta_{3} {reduction of three exacerbations}_{\mathrm{nsj}} + \beta_{4}{{mild side effects}_{\mathrm{nsj}} + \beta_{5} moderate to severe side effects}_{\mathrm{nsj}}+ \beta_{6}\mathrm{multidose}{}_{\mathrm{nsj}}+ \beta_{7} {low GWP}_{\mathrm{nsj}}+\beta_{8} {low GWP}_{\mathrm{nsj}} high\_work\_experience+ \beta_{9} {low GWP}_{\mathrm{nsj}} \mathrm{GP} + \beta_{10} {low GWP}_{\mathrm{nsj}} environment\_member$$

where $V_{nsj}$ is the observed utility of participant *n* for choice set *s* for alternative *j*;

alt is either of the two alternatives;

$\beta_{0}$ is the alternative specific constant;

$\beta_{1}$ is the linearly estimated coefficient of out of pocket costs;

$\beta_{2-3}$ are coefficients of reduction of two exacerbations or three exacerbations in the next year, as compared to the reference level of reduction in one exacerbation in the next year;

$\beta_{4-5}$ are coefficients of mild side effects and moderate to severe side effects, as compared to the reference level of no side effects.

$\beta_{6}$ is the coefficient of a multidose system, as compared to the reference level of unidose system;

$\beta_{7}$ is the coefficient of low global warming potential inhaler, as compared to the reference level of high global warming potential inhaler;

$\beta_{8}$ is the coefficient of low global warming potential inhaler, as compared to the reference level of high global warming potential inhaler, with the following interaction term to assess the systematic preference: > 10 years working in general practice as compared to ≤ 10 years working in general practice;

$\beta_{9}$ is the coefficient of low global warming potential inhaler, as compared to the reference level of high global warming potential inhaler, with the following interaction term to assess the systematic preference: GP as compared to nurse practitioner or nurse specialist;

$\beta_{10}$ is the coefficient of low global warming potential inhaler, as compared to the reference level of high global warming potential inhaler, with the following interaction term to assess the systematic preference: member of organisation or network for environmentally friendly healthcare as compared to not being a member.
