# Supplementary material for: The role of environmental impact in healthcare providers’ choices of inhalers for treatment of asthma and COPD: a discrete choice experiment
Source: BMC Prim Care. 2025 Sep 3;26:278. doi: 10.1186/s12875-025-02941-8 (PMC12406421; doi:10.1186/s12875-025-02941-8)
Supplement: Supplementary file 4 — Supplementary Material 4. [file 12875_2025_2941_MOESM4_ESM.docx]

**SUPPLEMENTARY FILE 4**

**Marginal rate of substitution calculation**

Willingness to pay was calculated for an inhaler without greenhouse gases using the following equation:

$$Willingness to pay= \frac{-\beta2}{\beta1}$$

The coefficients represent how much a participant is willing to pay for one unit change (or to change from one level to another) in an attribute, and are calculated by the ratio of the coefficient for attribute ‘GWP’ (β2) to the coefficient of attribute ‘out of pocket costs’ (β1).

Willingness to accept in number of reduction of exacerbation per year was calculated for an inhaler with greenhouse gases using the following equation:

$$Willingness to accept= \frac{-\beta2}{\beta1}$$

The coefficients represent how much a participant is willing to accept for one unit change (or to change from one level to another) in an attribute, and are calculated by the ratio of the coefficient for attribute ‘GWP’ (β2) to the coefficient of attribute ‘reduction in number of exacerbations’ (β1).
