# Supplementary material for: The role of environmental impact in healthcare providers’ choices of inhalers for treatment of asthma and COPD: a discrete choice experiment
Source: BMC Prim Care. 2025 Sep 3;26:278. doi: 10.1186/s12875-025-02941-8 (PMC12406421; doi:10.1186/s12875-025-02941-8)
Supplement: Supplementary file 5 — Supplementary Material 5. [file 12875_2025_2941_MOESM5_ESM.docx]

**SUPPLEMENTARY FILE 5**

**GPs’ (n=38) attitudinal questions towards climate change**

**Nurse specialists’/nurse practitioners’ (n=38) attitudinal questions towards climate change**
