# Supplementary material for: The role of environmental impact in healthcare providers’ choices of inhalers for treatment of asthma and COPD: a discrete choice experiment
Source: BMC Prim Care. 2025 Sep 3;26:278. doi: 10.1186/s12875-025-02941-8 (PMC12406421; doi:10.1186/s12875-025-02941-8)
Supplement: Supplementary file 6 — Supplementary Material 6. [file 12875_2025_2941_MOESM6_ESM.docx]

**SUPPLEMENTARY FILE 6**

**Results of the multinomial logit model in utilities**

|  | **Model A**** | | **Model B**** | |
| --- | --- | --- | --- | --- |
| **Attribute levels** | **Utility** | **(95%CI)** | **Utility** | **(95%CI)** |
| ASC* | **0.29** | **(0.09; 0.48)** | **0.28** | **(0.011; 0.44)** |
| Out of pocket costs per year (per €) | **-0.30** | **(-0.48; -0.13)** | **-0.32** | **(-0.51; -0.14)** |
| Reduction in number of exacerbations per year (per one exacerbation) | **0.91** | **(0.73; 1.08)** | **0.92** | **(0.73; 1.11)** |
| *Multidose/unidose system* |  |  |  |  |
| Unidose system *(reference level)* | 0.00 | - | 0.00 | - |
| Multidose system | **0.29** | **(0.02; 0.56)** | **0.32** | **(0.02; 0.61)** |
| *Risk of side effects* |  |  |  |  |
| No side effects *(reference level)* | 0.00 | - | 0.00 | - |
| Mild side effects | **-0.44** | **(-0.84; 0.04)** | **-0.42** | **(-0.84; -0.01)** |
| Moderate to severe side effects | **-2.32** | **(-2.71; -1.94)** | **-2.36** | **(-2.79; -1.92)** |
| *Impact on CO₂ emissions* |  |  |  |  |
| High GWP *(reference level)* | 0.00 | - | 0.00 | - |
| Low GWP | **1.22** | **(0.94; 1.50)** | 0.14 | (-0.04; 0.31) |
| *Systemic preference heterogeneity* |  |  |  |  |
| > 10 years working in general practice x low GWP | - |  | 0.46 | (-0.33; 1.24) |
| GP (excl. nurse practitioner/specialist) x low GWP | - |  | **0.91** | **(0.41; 1.68)** |
| Member of organisation or network for environmentally friendly healthcare x low GWP | - |  | -0.14 | (-1.06; 0.78) |
| *AIC* | 672.79 |  | 616.58 |  |

**Bold**: Statistically significance at 5% level.

**Abbreviations:** CI=confidence interval; ASC=alternative specific constant; GP=general practitioner; AIC=Akaike Information Criterion.

**Notes.** For a correct interpretation of the results, it is important to notice the different units of measurement, e.g. out of pocket costs is a continuous variable that is measured per euro, whereas multidose system is a categorical variable that are compared to their reference level. *The alternative specific constant (ASC) took the left-right bias of the choice processes of the participants (i.e. participants always choosing option A or B) into account. **Model A includes the results of the multinomial logit model. ***Model B includes the results of the multinomial logit model plus systematic preference heterogeneity with interaction terms.
